# Supplementary figures and images for: Genetic identification of a common collagen disease in Puerto Ricans via identity-by-descent mapping in a health system
Source: eLife. 2017 Sep 12;6:e25060. doi: 10.7554/eLife.25060 (PMC5595434; doi:10.7554/eLife.25060)

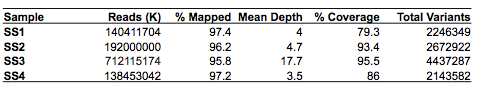

Supplement: Supplementary file 1. [file elife-25060-supp1.docx]

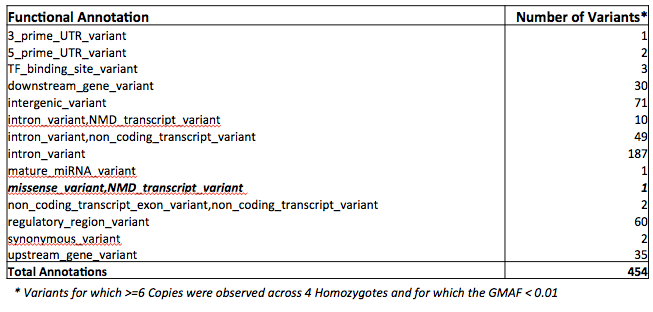

Supplement: Supplementary file 2. [file elife-25060-supp2.docx]

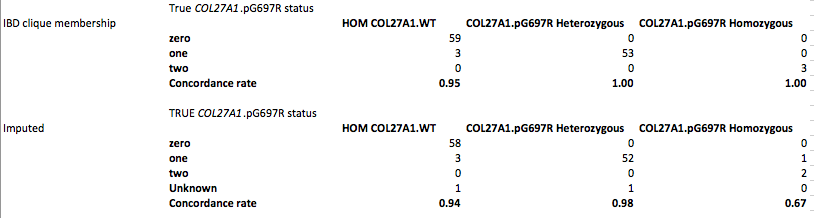

Supplement: Supplementary file 3. [file elife-25060-supp3.docx]
