# Supplementary material for: Genetic identification of a common collagen disease in Puerto Ricans via identity-by-descent mapping in a health system
Source: eLife. 2017 Sep 12;6:e25060. doi: 10.7554/eLife.25060 (PMC5595434; doi:10.7554/eLife.25060)
Supplement: Supplementary file 2 [file elife-25060-supp4.docx]

Region	Population	N	# Obs hets	# Obs hom	Allele Freq (q)	Carrier Rate
**Africa**	*Bantu (Kenya)*	*18*	*0*	*0*	*0*	-
	Mandenka	*21*	*0*	*0*	*0*	-
	Yoruban	*22*	*0*	*0*	*0*	-
	San	*12*	*0*	*0*	*0*	-
	Mbuti Pygmy	*10*	*0*	*0*	*0*	-
	Biaka Pygmy	*21*	*0*	*0*	*0*	-
	Khomani/Nama	45	0	0	0	-
	Senegal	31	0	0	0	-
	Ghana	30	0	0	0	-
	IvoryCoast	21	0	0	0	-
	Guinea	9	0	0	0	-
	Nigeria	19	0	0	0	-
	**Total**	**259**				
**Europe**	*Orcadian*	*13*	*0*	*0*	*0*	-
	Adygei	*149*	*0*	*0*	*0*	-
	Russian	*25*	*0*	*0*	*0*	-
	Basque	*23*	*0*	*0*	*0*	-
	French	*28*	*0*	*0*	*0*	-
	Italian	*12*	*0*	*0*	*0*	-
	Sardinian	*25*	*0*	*0*	*0*	-
	Tuscan	*7*	*0*	*0*	*0*	-
	Total	***282***				
**West Asia**	*Mozabite*	*29*	*0*	*0*	*0*	-
	Bedouin	*46*	*0*	*0*	*0*	-
	Druze	*40*	*0*	*0*	*0*	-
	Palestinian	*41*	*0*	*0*	*0*	-
	Total	***156***				
**Central and South Asia**	*Balochi*	*24*	*0*	*0*	*0*	-
	Brahui	*25*	*0*	*0*	*0*	-
	Makrani	*25*	*0*	*0*	*0*	-
	Sindhi	*24*	*0*	*0*	*0*	-
	Pathan	*24*	*0*	*0*	*0*	-
	Burusho	*25*	*0*	*0*	*0*	-
	Hazara	*19*	*0*	*0*	*0*	-
	Uygur	*9*	*0*	*0*	*0*	-
	Kalash	*21*	*0*	*0*	*0*	-
	Pakistan	68	0	0	0	-
	India	225	0	0	0	-
	Bangladesh	158	0	0	0	-
	SriLanka	10	0	0	0	-
	**Total**	**657**				

Region	Population	N	# Obs hets	# Obs hom	Allele Freq (q)	Carrier Rate
**East Asia**	*Han*	*34*	*0*	*0*	*0*	-
	Han (North China)	*10*	*0*	*0*	*0*	-
	Dai	*10*	*0*	*0*	*0*	-
	Daur	*10*	*0*	*0*	*0*	-
	Hezhen	*8*	*0*	*0*	*0*	-
	Lahu	*8*	*0*	*0*	*0*	-
	Miao	*10*	*0*	*0*	*0*	-
	Oroqen	*9*	*0*	*0*	*0*	-
	She	*9*	*0*	*0*	*0*	-
	Tujia	*10*	*0*	*0*	*0*	-
	Tu	*10*	*0*	*0*	*0*	-
	Xibo	*9*	*0*	*0*	*0*	-
	Yi	*10*	*0*	*0*	*0*	-
	Mongola	*10*	*0*	*0*	*0*	-
	Naxi	*8*	*0*	*0*	*0*	-
	Cambodian	*10*	*0*	*0*	*0*	-
	Japanese	*29*	*0*	*0*	*0*	-
	Yakut	*25*	*0*	*0*	*0*	-
	Asian (USA)	309	0	0	0	-
	Philippines	211	0	0	0	-
	China	163	0	0	0	-
	Japan	85	0	0	0	-
	Korea	63	0	0	0	-
	Hong Kong	25	0	0	0	-
	Taiwan	25	0	0	0	-
	Vietnam	12	0	0	0	-
	Thailand	11	0	0	0	-
	Japanese (Hawaii)	4196	0	0	0	-
	**Total**	**5329**				
**Oceania**	*Melanesian*	*10*	*0*	*0*	*0*	-
	Papuan	*17*	*0*	*0*	*0*	-
	Native Hawaiian (USA)	4179	1	0	1.2x10-4	1:4179
	**Total**	**4206**				

Region	Population	N	# Obs hets	# Obs hom	Allele Freq (q)	Carrier Rate
**Americas**	*Karitiana*	*8*	*0*	*0*	*0*	-
	Surui	*4*	*0*	*0*	*0*	-
	Colombian	*6*	*0*	*0*	*0*	-
	Maya	*21*	*0*	*0*	*0*	-
	Pima	*11*	*0*	*0*	*0*	-
	Andean	303	0	0	0	-
	Bari	24	0	0	0	-
	Indigenous Honduran	66	0	0	0	-
	Oaxacan	84	0	0	0	-
	Warao	22	0	0	0	-
	Yukpa	24	0	0	0	-
	Native American (USA)	681	0	0	0	-
	Hispanic/Latino (USA)	4797	64	2	0.007	1:72
	Hispanic/Latino (Mixed/Other)	1325	0	0	0	-
	Mexico	6416	0	0	0	-
	Puerto Rico	5158	102	3	0.01	1:51
	Dominican Republic	2985	4	0	6.7x10-4	1:746
	Cuba	2426	0	0	0	-
	St. Thomas	9	1	0	0.11	1:9
	Jamaica	394	0	0	0	-
	Trinidad andTobago	172	0	0	0	-
	Haiti	166	0	0	0	-
	St.Kitts and Nevis	9	0	0	0	-
	Barbados	46	0	0	0	-
	Antigua and Barbuda	18	0	0	0	-
	Grenada	13	0	0	0	-
	Central American	1310	0	0	0	-
	El Salvador	76	0	0	0	-
	Guatemala	40	0	0	0	-
	Panama	31	0	0	0	-
	Honduras	132	0	0	0	-
	South American	782	0	0	0	-
	Ecuador	434	0	0	0	-
	Colombia	113	0	0	0	-
	Peru	124	0	0	0	-
	Argentina	19	0	0	0	-
	Venezuela	19	0	0	0	-
	Chile	16	0	0	0	-
	Brazil	15	0	0	0	-
	Costa Rica	13	0	0	0	-
	Guyana	313	0	0	0	-
	Belize	29	0	0	0	-
	African-American	17,773	1	0	2.8x10-5	1:17,773
	**Total**	**46427**				
